# Structure- and context-based analysis of the GxGYxYP family reveals a new putative class of Glycoside Hydrolase

**DOI:** 10.1186/1471-2105-15-196

**Published:** 2014-06-17

**Authors:** Daniel J Rigden, Ruth Y Eberhardt, Harry J Gilbert, Qingping Xu, Yuanyuan Chang, Adam Godzik

**Affiliations:** 1 Institute of Integrative Biology, University of Liverpool, Liverpool, UK; 2 Wellcome Trust Sanger Institute, Wellcome Trust Genome Campus, Hinxton, Cambridgeshire CB10 1SA, UK; 3 European Molecular Biology Laboratory, European Bioinformatics Institute, Hinxton, Cambridgeshire CB10 1SD, UK; 4 Institute for Cell and Molecular Biosciences, The Medical School, Newcastle University, Framlington Place, Newcastle Upon Tyne NE2 4HH, UK; 5 Joint Center for Structural Genomics, Stanford Synchrotron Radiation Lightsource, SLAC National Accelerator Laboratory, Menlo Park CA 94025, USA; 6 Joint Center for Structural Genomics, Program on Bioinformatics and Systems Biology, Sanford-Burnham Medical Research Institute, La Jolla CA 92037, USA; 7 Joint Center for Structural Genomics, Center for Research in Biological Systems, University of California, San Diego, La Jolla CA 92093, USA

**Keywords:** Carbohydrate metabolism, Glycoside hydrolase, Polysaccharide Utilization Locus, PUL, Protein function prediction, JCSG, 3D structure, Protein family, Gut microbiota

## Abstract

**Background:**

Gut microbiome metagenomics has revealed many protein families and domains found largely or exclusively in that environment. Proteins containing the GxGYxYP domain are over-represented in the gut microbiota, and are found in Polysaccharide Utilization Loci in the gut symbiont *Bacteroides thetaiotaomicron*, suggesting their involvement in polysaccharide metabolism, but little else is known of the function of this domain.

**Results:**

Genomic context and domain architecture analyses support a role for the GxGYxYP domain in carbohydrate metabolism. Sparse occurrences in eukaryotes are the result of lateral gene transfer. The structure of the GxGYxYP domain-containing protein encoded by the BT2193 locus reveals two structural domains, the first composed of three divergent repeats with no recognisable homology to previously solved structures, the second a more familiar seven-stranded β/α barrel. Structure-based analyses including conservation mapping localise a presumed functional site to a cleft between the two domains of BT2193. Matching to a catalytic site template from a GH9 cellulase and other analyses point to a putative catalytic triad composed of Glu272, Asp331 and Asp333.

**Conclusions:**

We suggest that GxGYxYP-containing proteins constitute a novel glycoside hydrolase family of as yet unknown specificity.

## Background

The era of pyrosequencing has shed light on new areas of the protein sequence universe, revealing new domains and expanding membership of existing domains [1,2]. One particularly fruitful environment has been the mammalian gut microbiome that has been shown to correlate with, and even directly influence, several human disease states [3,4]. There is therefore an urgent need to characterise the structure and function of domains discovered in gut metagenome data [5,6], especially those found to be particularly over-represented in gut microbes [7]: many of these are likely to be involved in interaction with the host and potentially, therefore, targets of interest for future pharmacological intervention.

*Bacteroides* is a genus of Gram-negative bacteria, particularly prominent in the distal gut of mammals, including humans, and typically making up to 30% of the microbiota [8]. A particularly well-studied *Bacteroides* species is *B. thetaiotaomicron* which is most notable for its sophisticated carbohydrate metabolism. This centres on 88 Polysaccharide Utilization Loci (PUL), accounting for almost a fifth of its genome, each one coding for a set of enzymes - hydrolases, esterases, lyases etc. - collectively capable of digesting a specific carbohydrate, along with corresponding signalling and transport proteins [9]. Notably, these target polysaccharides include many that are indigestible to the host, so that *B. thetaiotaomicron* provides additional advantage to the host making their relation mutualistic if not symbiotic. However, in other circumstances, *Bacteroides* and other bacteria can produce enzymes that degrade the carbohydrate components of host cell surface glycoproteins such as mucin [10]. Although, with the benefit of the Carbohydrate-Active enZYmes (CAZy) database [11], many proteins encoded by PULs can be straightforwardly and reliably assigned functions, others are presently defined only as hypothetical proteins showing that further groups of proteins involved in carbohydrate metabolism remain to be characterised.

Here we apply wide range of bioinformatics methods, including structure-based analyses of a newly determined crystal structure, to predict a function for the GxGYxYP domain, found in four PULs in *B. thetaiotaomicron*. We show that further genomic context and domain architecture information support the broad implication of the domain in carbohydrate metabolism. The functional site of the GxGYxYP domain is strongly predicted, by multiple methods, to lie between the two structural domains revealed by the crystal structure of BT2193 (GxGYxYP_N [Pfam:PF16216] and GxGYxYP_C [Pfam:PF14323]). Three conserved acidic residues are arranged in a similar manner as those comprising the catalytic site of unrelated cellulases and suggest that the GxGYxYP domain defines a new family of glycoside hydrolase (GH).

## Results

### Phylogenetic distribution

Interestingly, proteins bearing the GxGYxYP domain are highly over-represented in human gut metagenomics samples: there are currently around seven times as many such sequences in the MetaHIT database [7] as in UniProt [12]. The average ratio for MetaHIT:UniProt representation for a Pfam domain is 7:100 (unpublished data) showing that the over-representation is around 100-fold. This places it at position 117 in a ranking of MetaHIT over-represented Pfam domains. Interestingly, proteins from this domain are also present, but not so prevalent, in metagenomics samples representing other environments, such as ocean or soil.

**Figure 1:**
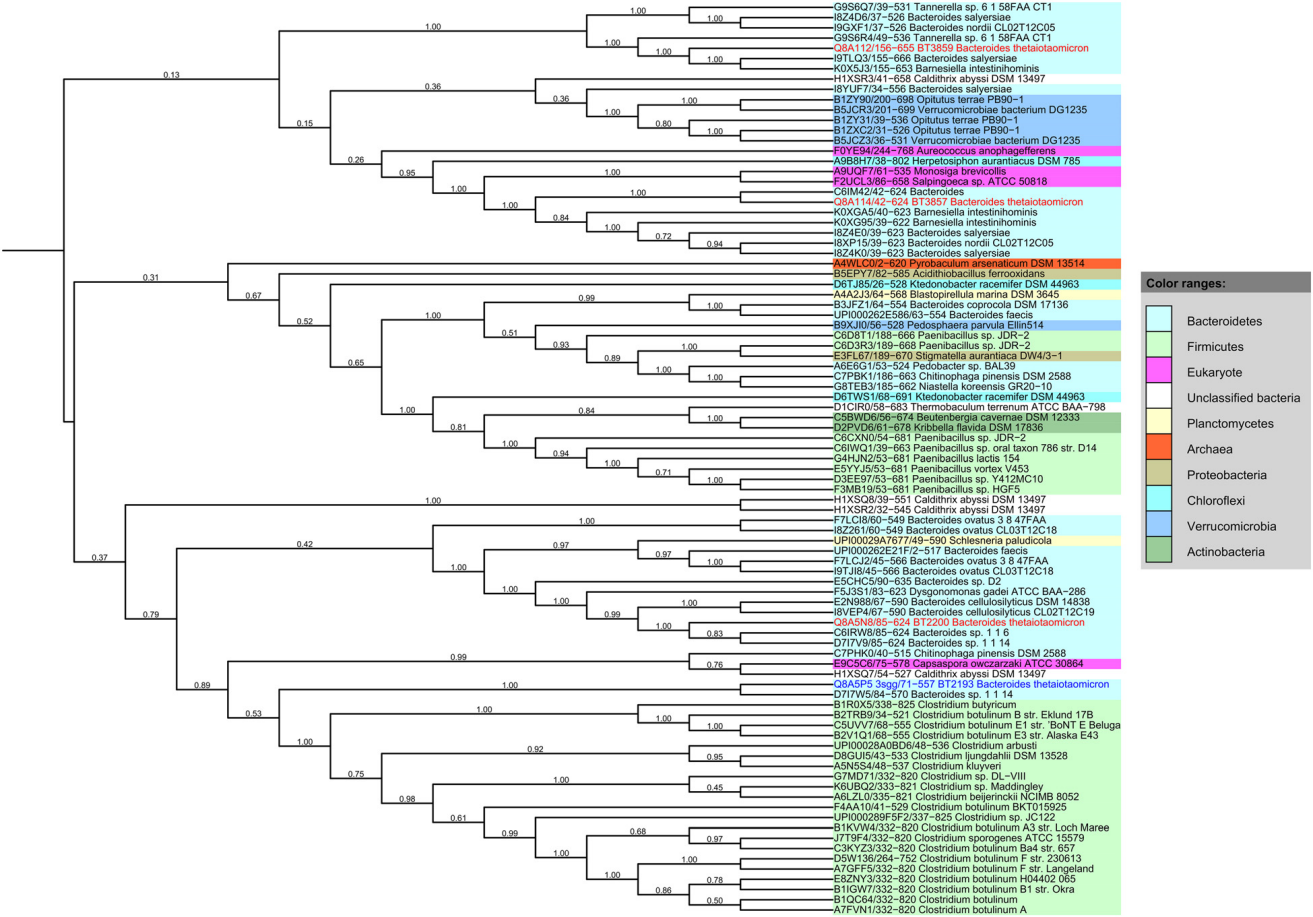
**Bootstrapped neighbour-joining unrooted phylogenetic tree of GxGYxYP proteins.** The tree was calculated using the GxGYxYP region alone as described in Methods with MEGA 5 [13]. Bootstrapping values are given to the left of the node in question and are on a scale of 0–1. For each sequence the accession, residue range of the GxGYxYP domain and species name are given. Loci for *B. thetaiotaomicron* sequences discussed in the text are also given and those sequences coloured blue (BT2193 whose structure is reported here) or red.

The distribution of a protein family often provides clues as to its function. We therefore analysed the set of species bearing GxGYxYP domains and carried out bootstrapped phylogenetic analysis on the set of full-length GxGYxYP obtained by database searching. The distribution of GxGYxYP-containing species is largely bacterial, with Proteobacteria, Firmicutes and Actinobacteria all represented. Interestingly, however, the domain is seen sporadically in diverse eukaryotes such as *Capsaspora owczarzaki* and the choanoflagellates *Monosiga brevicolis* and *Salpingoeca* strain ATCC 50818. There is a single archaeal sequence, from *Pyrobaculum arsenaticum*, in the present database. The single sequence from moss (*Physcomitrella patens* subsp. patens) [UniProt:A9U7X7] is annotated as a fragment. The genome of moss is a draft genome and limited extension of the protein sequence is possible at the N-terminus. This extension is most similar to sequences from *Paenibacillus* so it is possible that the moss sequence is a contaminant. The sporadic eukaryotic distribution does not suggest that this domain was found in the common ancestor of bacteria and eukaryotes. Indeed, phylogenetic analysis provides strong evidence for acquisition of the domain by eukaryotes through multiple instances of lateral gene transfer. As Figure 1 shows, the Monosiga and Salpingoeca sequences are found in a well-supported clade bounded by the *Herpetosiphon aurantiacus* sequence. Similarly, the Capsaspora sequence resides in a clade bounded by the bacterial *Chitinophaga pinensis* sequence.

### Gene context

**Figure 2:**
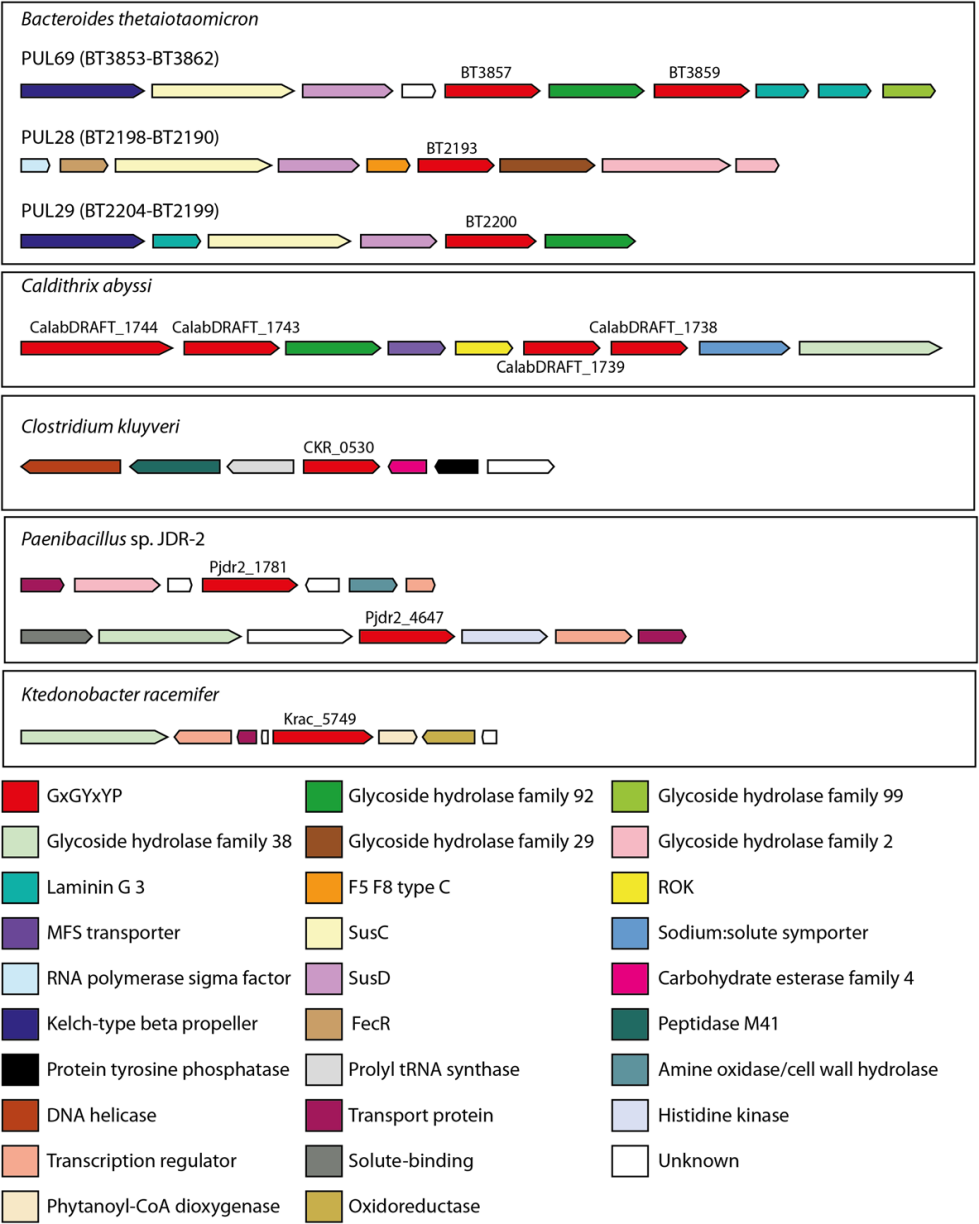
**Genomic context of selected genes encoding GxGYxYP family proteins.** Genomic context was determined using MicrobesOnline [14]. Genes are coloured according to protein family membership, which was determined using Pfam [15] and InterPro [16].

One of the species with the largest number of GxGYxYP proteins is *B. thetaiotamicron*, a prominent commensal gut bacterium. Along with other *Bacteroides* species it can process a large number of different polysaccharides, both plant compounds eaten by the host and host-derived complex carbohydrates. Upon exposure to carbohydrates, appropriate sets of metabolic enzymes are induced from genes arranged in Polysaccharide Utilization Loci (PULs). Intriguingly, genes encoding GxGYxYP proteins in *Bacteroides thetaiotamicron* are found in PULs, implicating them broadly in carbohydrate metabolism (see Figure 2). Genes for two, BT3857 and BT3859, are found in PUL 69, characterised as being responsive to α-mannans which is used as a nutrient by the bacterium. Two others, BT2193 and BT2200, are found in neighbouring PULs, numbered 28 and 29, respectively, each of unknown target polysaccharide. BT2193 is followed by a gene encoding α-L-fucosidase (glycoside hydrolase family 29) and a member of glycoside hydrolase family 2, a family reported to have β-galactosidase, β-mannosidase, β-glucuronidase, mannosylglycoprotein endo-β-mannosidase and exo-β-glucosaminidase activities. BT2200 is followed by a gene encoding an α-1,2-mannosidase belonging to glycoside hydrolase family 92.

*Caldithrix abyssi,* an anaerobic bacterium found in deep-sea hydrothermal chimneys and representing a new, as yet unclassified bacterial group*,* also has four GxGYxYP proteins (see Figure 2). The genes encoding these proteins are arranged in two pairs, separated by genes encoding three other proteins: an α-1,2-mannosidase belonging to glycoside hydrolase family 92, an MFS transporter (which may be a sugar transport protein [17]) and a ROK family protein. A gene encoding an α-mannosidase belonging to glycoside hydrolase family 38 is also found near to these genes. GxGYxYP is also found near to glycoside hydrolases and other enzymes involved in polysaccharide metabolism in several other species; some examples are given in Figure 2. This is also suggestive of a role in polysaccharide metabolism.

### Domain architectures

**Figure 3:**
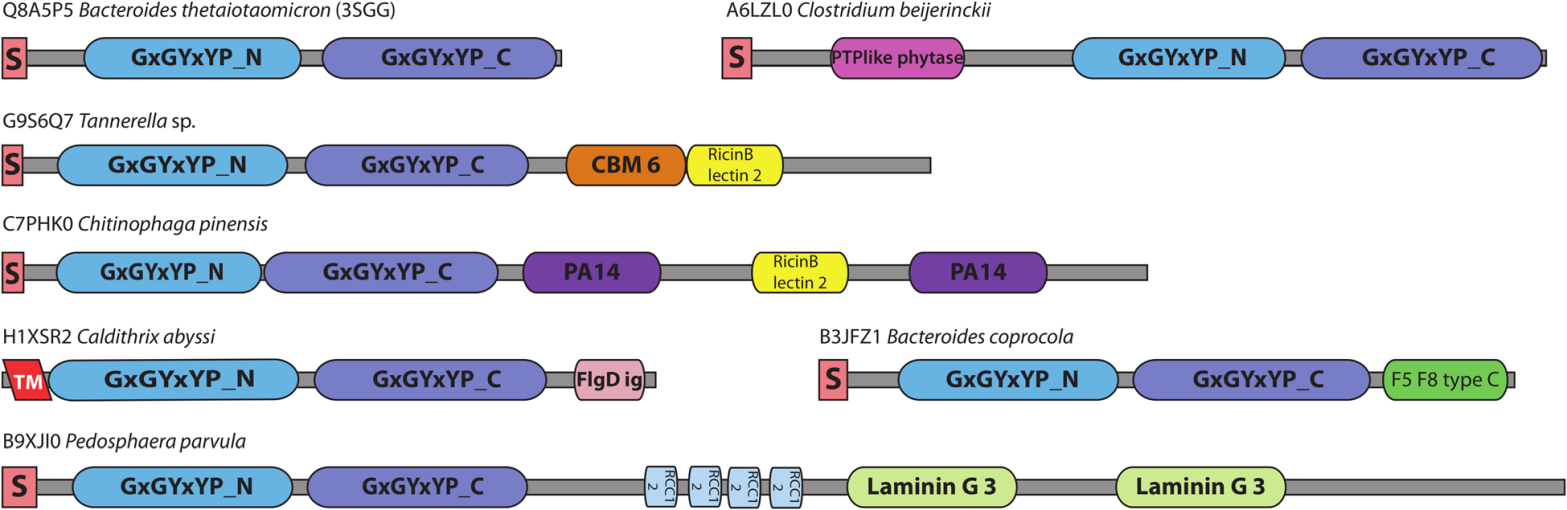
**Domain architectures of selected GxGYxYP family members.** Domain architectures were predicted by Pfam [15]. Signal peptides and transmembrane regions were predicted using Phobius [32].

Protein domains found in the same molecule are often functionally related [18] so study of the domains which are found to co-occur with GxGYxYP may provide important clues to its function. The N-terminal and C-terminal domains, GxGYxYP_N [Pfam:PF16216] and GxGYxYP_C [Pfam:PF14323] are always found associated with each other. In addition to this several other protein domains are found in proteins containing GxGYxYP, the majority having a role in carbohydrate binding or recognition (see Figure 3). The carbohydrate-binding module CBM6 [Pfam:PF03422] has been shown to bind to a variety of polysaccharides including xylan, β-1,3-glucans, β-1,4-glucans and β-1,3-β-1,4-mixed linkage glucans [19-21]. The F5/8 type C domain [Pfam:PF00754] is a carbohydrate-binding module belonging to the Galactose-binding domain-like superfamily. It is classified by CAZy as CBM32 and can bind to galactose and N-acetylgalactosamine [22]. A recently characterised mucin-degrading enzyme from *Bacteroides thetaiotaomicron*[23] carries this domain and a BACON domain [6], both likely to target carbohydrate substructures in the substrate. The same work noted that members of the protease domain family characterised therein bearing the CBM32 domain were predominantly those from organisms associated with the mucosal surface. The Ricin-type beta-trefoil lectin domain (RicinB_lectin_2 [Pfam:PF14200]) has been found to bind the linear trisaccharide Gal-α-(1,3)-Gal-β-(1,4)-GlcNAc, sialylated glycans terminating with Neu5Ac-α-(2–6)-Gal, N,N'-diacetyllactosediamine and GalNAc-containing oligosaccharides [24-27]. Another lectin found in association with GxGYxYP is a member of the concanavalin A-like lectin/glucanases superfamily, Laminin_G_3 [Pfam:PF13385], this domain is thought to play a role in carbohydrate recognition [28]. The PA14 domain [Pfam:PF07691] is often found in glycosyl hydrolases and glycosyltransferases where it is involved in carbohydrate-binding and recognition and determination of substrate-specificity [29-31]. This co-occurrence with carbohydrate-binding and recognition domains suggests a carbohydrate-related function for GxGYxYP.

The only catalytic domain found fused to GxGYxYP is the phytase domain [Pfam:PF14566] found at the C-terminus of some Clostridial phytases. Phytases hydrolyse phytate (found in plant seeds) resulting in the release of phosphate [33]. This domain is presumably the origin of the hydrolase annotations of some GxGYxYP proteins.

### Crystal structure determination

The crystal structure of the GxGYxYP domain-containing protein (encoded by locus BT2193) from *Bacteroides thetaiotaomicron* VPI-5482 was determined to 1.25 Å resolution by the MAD method. Data collection, model and refinement statistics are summarized in Additional file 1: Table S1. The final model includes one molecule (residues 23–557), five glycerol and 550 water molecules in the asymmetric unit. Gly0 (which remained at the N-terminus after cleavage of the expression/purification tag), and the region from Ala23 to Gly45 were disordered and not modeled. All the side chains were fully modeled because of the high quality of electron density. The Matthews coefficient (Vm; [34]) is 2.16 Å^3^ Da^-1^ and the estimated solvent content is 43.1%. The Ramachandran plot produced by MolProbity [35] shows that 98.0% of the residues are in favored regions, with no outliers.

### Structure description

**Figure 4:**
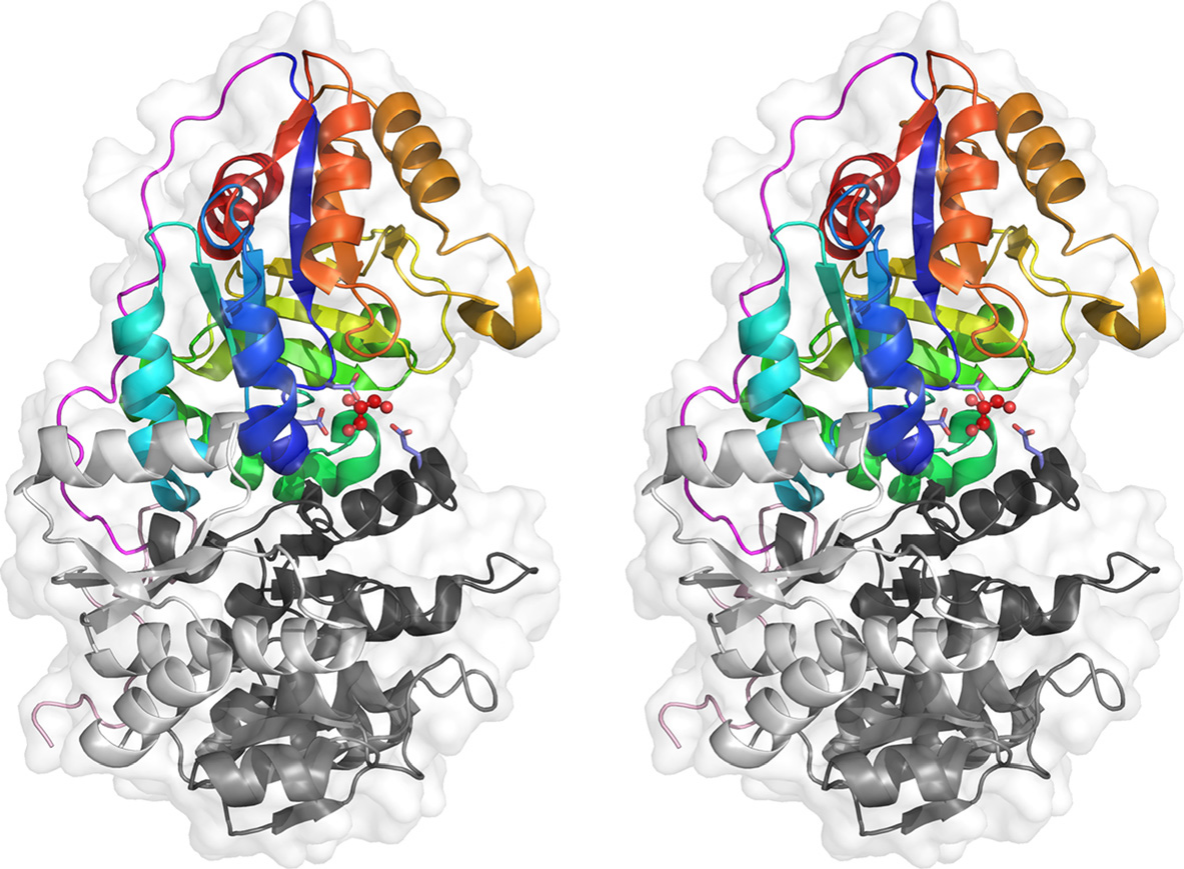
**Cross-eyed stereo cartoon representation of the BT2193 structure.** It is coloured light pink (largely irregular extreme N-terminal region), shades of grey in the N-terminal domain (light grey repeat 1, mid-grey repeat 2, dark grey repeat 3), magenta (inter-domain linker) and thereafter as a spectrum (blue start to red end in the C-terminal barrel domain). The putative catalytic triad (see later) is shown as sticks and a glycerol molecule bound as ball and stick. A semi-transparent surface helps visualise the presence of the site and bound glycerol in a cleft between the two domains.

The structure of BT2193 GxGYxYP structure reveals two domains, an N-terminal domain with alternating α and β structure (residues 46–299) (GxGYxYP_N, [Pfam:PF16216]) and a C-terminal 7-stranded β/α barrel domain (residues 321 to 557) (GxGYxYP_C, [Pfam:PF14323]) (Figure 4).

They are connected by an extended linker region, lacking regular secondary structure, which lies across the top of the barrel. Querying the PDB for similar structures with the full-length structure yields results monopolised by the C-terminal domain. It exhibits strong structural similarity (Z-scores >13) with 7-stranded barrels found in allantoinases eg [PDB:3 cl6], polysaccharide deacetylases eg [PDB:3rxz] and glucanotransferases eg [PDB:1k1x]. However, the sequence identity shared by matching regions did not exceed 15% and was generally much lower. Furthermore, analysis of the structure alignments revealed that in no case were key catalytic residues found in matching structures present in BT2193. Sequence-based analysis gave similar results. Thus, although families with barrel domains such as Carbohydrate Esterase family 4 were reliably matched by sensitive Hidden Markov Model comparisons using HHpred [36,37], sequence identities were very low, not exceeding 14%, and key catalytic determinants in the hits were not present in the BT2193 sequence. This clearly indicated that the GxGYxYP domain represented a new family, rather than a divergent branch of a known family.

**Figure 5:**
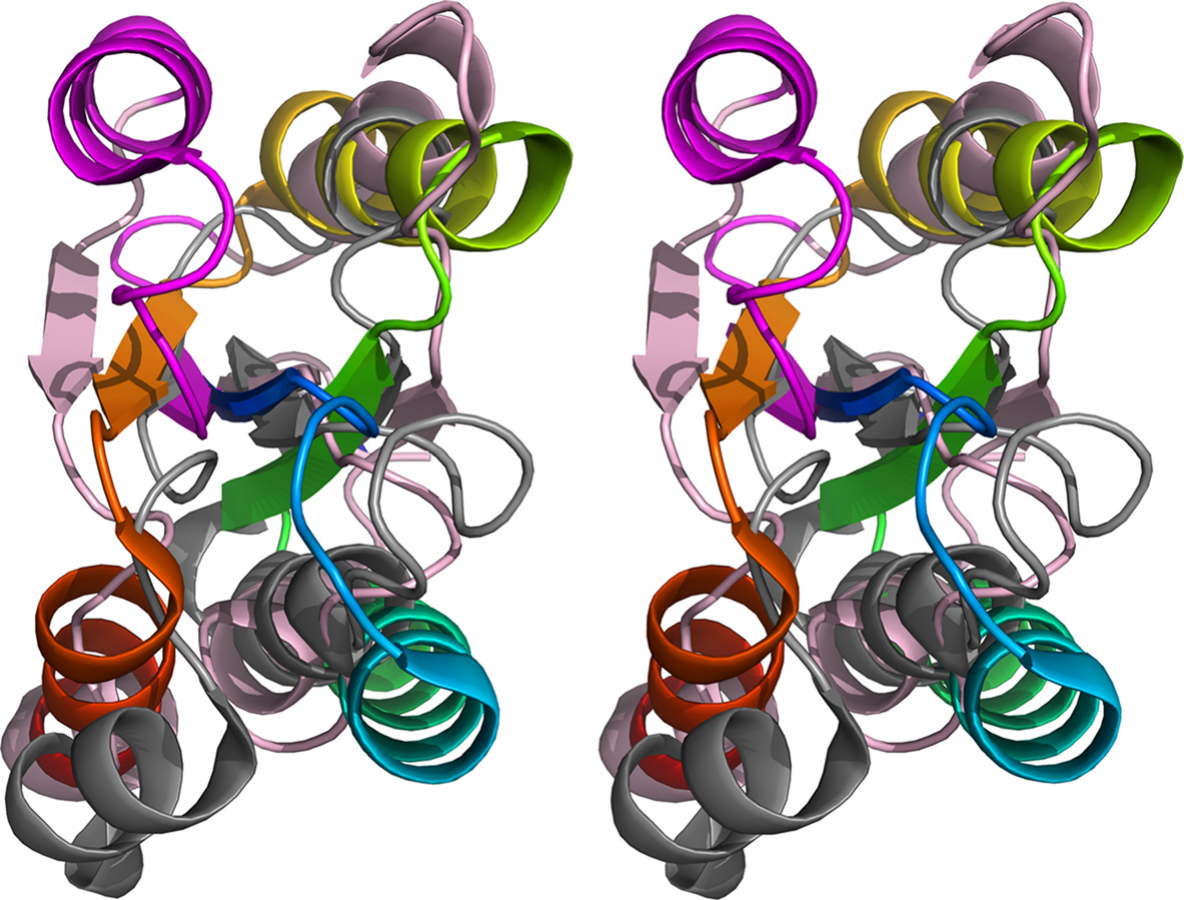
**Cross-eyed stereo view of the structural superposition of the three repeats in the N-terminal domain.** The superposition was made with PDBeFOLD [39]. The first repeat is coloured as a spectrum from blue (N-terminus) to red (C-terminus). The second repeat is uniformly grey. The third repeat is shown in light pink for its majority that is topologically the same as the first two repeats, and dark pink for its additional β/α motif that is defined as an insertion by the structural superposition.

In broad terms, the 7-stranded β/α barrel is weakly suggestive of a role for BT2193 in carbohydrate metabolism. The SCOP database [38], for example, divides these barrel structures into three superfamilies. One contains known and predicted GH family 6 [11] cellulases. The second contains GH family 38 enzymes - which include α-mannosidases that attack eukaryotic N-glycans, glucanotransferases and polysaccharide deacetylases - but also allantoinases and proteins of unknown function. The third superfamily contains predicted phosphoesterases and subunits of ribonuclease complexes.Examination of the N-terminal domain revealed the presence, after a largely irregular short stretch from positions 46–65 containing a β-hairpin, of three β/α repeat subdomains comprising residues 66–141, 142–210 and 211–299, respectively. Each contains a central, twisted β-sheet with helices packed on both sides and, although the third repeat contains a four-stranded sheet compared to the three-stranded sheets of the first two, this repeating nature is suggestive of an origin in tandem duplication. Structural alignment of the three repeats illustrates the topological identity of the first two repeats. The same alignment shows by defining the third β/α motif of the four in the third repeat as an insertion the remainder has the same topology as the entirety of the first two repeats (Figure 5). The alignment results in matching of 53 residues across the three repeats with an overall RMSD of 3.71 Å but insignificant sequence identities of 6–8%.

Superficially, these subdomains resemble the Rossmann fold yet that structure was not prominently featured in DALI results for the third subdomain which shares its four β/α construction. The top hit for the third domain (with a Z score of 5.8) was in fact a periplasmic binding protein which contains a central six-stranded sheet. A minimal, artificial Rossmann fold [PDB:2kpo] was the top structural match (Z score 4.2) of the first subdomain yet there was again a fundamental mismatch between the three-stranded central sheet of the BT2193 sub-domain and the four-stranded Rossmann fold. Overall, the results reveal no clear homology between the repeating subdomain of the N-terminal domain and any known structure.

### Structure-based function prediction

**Figure 6:**
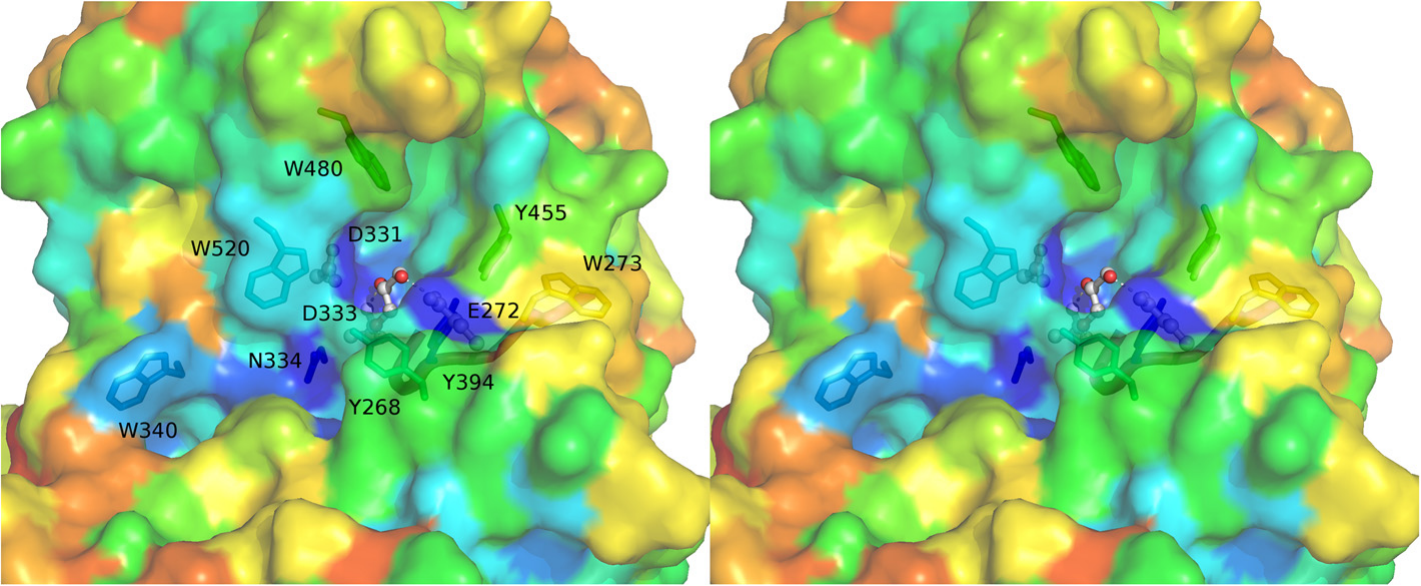
**Cross-eyed stereo view of the conserved cleft between the two domains in the BT2193 structure.** Surface and individual residues are coloured on a spectrum according to ConSurf [44] conservation values with blue indicating conservation and red its lack. Putative catalytic acidic residues are shown in ball and stick, as is the nearby bound glycerol molecules in white whose hydrogen bonds to the protein are shown as dotted lines. Surface-exposed Tyr and Trp residues, mainly well-conserved, and an additional highly conserved residue Asn334 and are shown in a stick representation. The region matching the domain-defining sequence motif GxGYxYP (391 GSSYIFP 397 here) is shown as a magenta tube and bears one of the conserved solvent-exposed aromatic residues, Tyr394.

Binding proteins typically interact with their ligands at their largest cavities [40]. Cavity analysis via Profunc [41] revealed that the largest cavity in the BT2193 structure lies between the two domains. Its volume is estimated at 2224 Å^3^, significantly larger than the next largest cavity with a volume of 1341 Å^3^. Within the cavity, Profunc also picks out a nest structure [42] from Asp331-Asp333. Associated with ion binding, these nests with characteristic local protein backbone structure are significantly associated with protein functional sites [42]. Also interestingly, the largest cavity contains one of five glycerol molecules bound to the protein, presumably deriving from the crystallisation conditions. The glycerol bound by the cavity hydrogen bonds to Asp333 and Glu272 side chains and additionally contacts Tyr394 (Figure 6). Fortuitously bound glycerol molecules often bind in carbohydrate binding sites of proteins. Sites determining functions shared across a protein family are also expected to be conserved in sequence [43]. Figure 6 shows that the inter-domain cleft bearing the nest structure is a major sequence conserved patch on the protein surface.

**Figure 7:**
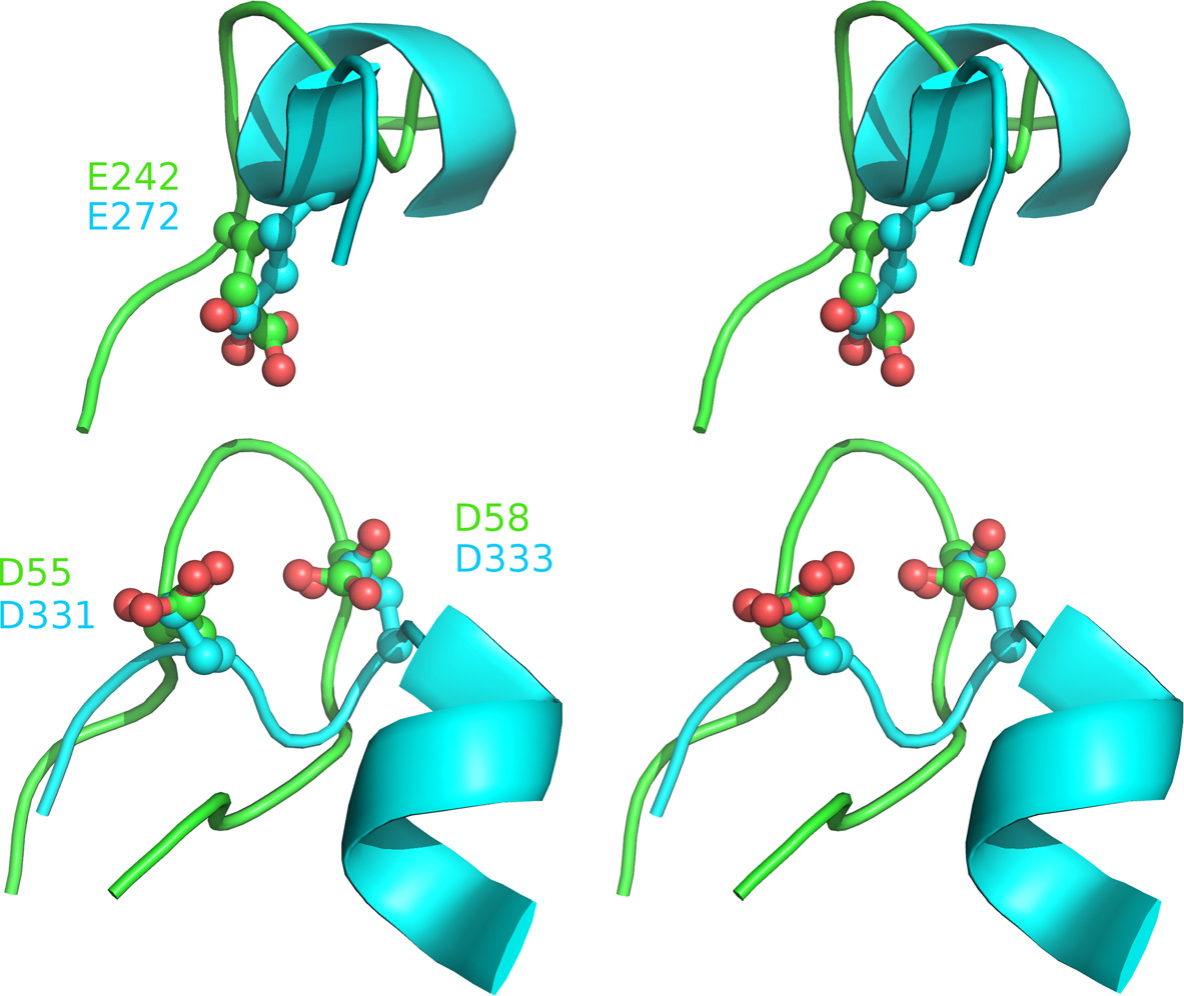
**Cross-eyed stereo figure illustrating the superposition of the catalytic triad of a GH9 cellulase from *Thermomonospora fusca ***[54]** (green; [PDB:1js4]) with the putative catalytic site identified here in the structure of BT2193 (cyan).** Some backbone context is shown to emphasise that the similarity arose by convergent evolution not homology.

A further powerful method for prediction of functional sites is picking out examples of local convergent structural evolution. For example, the Ser-His-Asp catalytic triad originally characterised in trypsin-like proteases has since been discovered, along with variant forms such as Ser-His-Glu, in diverse folds with protease, lipase, acyltransferase and general esterase activities eg [45-47]. More broadly, many papers have established that convergently evolved binding sites for a shared ligand such as ATP bear three-dimensional physicochemical similarities eg [48-50]. Predictive methods based on these observations centre on 3d matching of structural motifs or pocket characteristics. To this end the BT2193 structure was screened against the Catalytic Site Atlas database (CSA; [51]) at both the SPRITE [52] and PINTS [53] servers. Given the strong evidence (above) that the largest cleft harbours the functional site(s) of the GxGYxYP family, the results were browsed for significant hits involving residues in that cleft. Strikingly, each method produced a significant match against the CSA entry for a GH9 bacterial cellulase E4 [54] [PDB:1js4]. The cellulase catalytic site centres on three acidic residues Asp55, Asp58 and Glu424. SPRITE and PINTS superimpose BT2193 positions Asp333, Asp331 and Glu272, respectively, on the cellulase residues with rmsd values of 1.31 and 0.8 Å (Figure 7). Importantly, each of these three positions is highly conserved, though not invariant (Additional file 2: Figure S1) as would be expected if they formed an analogous catalytic site in the GxGYxYP family. It is worth noting that the three residues of the well-characterised cellulase catalytic site are also not invariant in a sequence alignment of the GH9 family, perhaps due to sporadic loss of catalysis but a maintained binding role in certain species. Note also that, in addition to the different sequential order of the (putative) catalytic triads, there is no structural resemblance between the cellulase and BT2193. The cellulase contains a six-hairpin α/α toroidal structure entirely forming the catalytic site. In contrast, the putative site in BT2193 lies between the C-terminal barrel domain, contributing Asp331 and Asp333, and the internally repeating N-terminal domain described earlier that contributes Glu272. Of the three acidic residues in BT2193, two were picked out as a nest structure and two contact bound glycerol (see above).

If the identified acidic residues in BT2193 were indeed a site for glycoside hydrolase activity then the expectation would be that neighbouring surfaces would bind other monosaccharides in the substrate conferring specificity and/or binding affinity. In glycosidases a minimum site would bind a disaccharide, but endo-acting glycanases, which cleave glycosidic linkages within polysaccharide chains, would bind more than one monosaccharide unit flanking both sides of the glycosidic bond to be hydrolysed. Crystal structures of the E4 cellulase, for example, reveal six monosaccharide-binding subsites [54]. These sites commonly contain otherwise unusual solvent-exposed aromatic amino acids [55], particularly tryptophan [56], since they form favourable hydrophobic interactions with the hydrophobic faces of cyclic saccharide structures [57] while also providing directional plasticity enabling a processive mode of action, when appropriate [58]. In the BT2193 structure, a number of such residues, mostly well-conserved, can be seen (Figure 6) providing further circumstantial support for a carbohydrate-binding function. Such aromatic residues are also found at protein-protein interfaces but the shape of the conserved patch - a cleft rather than a flat surface - supports carbohydrate binding over interaction with another protein. Interestingly, the ‘GxGYxYP’ motif (represented by 391GSSYIFP397 in BT2193), despite lying near to the putative catalytic site and containing aromatic residues, is almost entirely buried and only the side chain of Tyr394 is positioned where it may be available for interaction with substrate. Notably, an additional strongly conserved residue neighbouring the acidic triad is Asn334, also an amino acid strongly over-represented at carbohydrate-binding sites [56].

Taken together, these results are highly suggestive of a convergently evolved glycoside hydrolase catalytic site lying in the large, conserved inter-domain cleft. By analogy with the cellulase catalytic mechanism [54] it can be proposed that either Asp333 or Asp331 acts to deprotonate a water that would bind between the pair. Azide rescue experiments on GH9 cellulases show that Asp58 fulfils this role [59] so, although they relate to an analogous site, those data suggest that the structurally corresponding Asp333, may be considered the more likely catalytic base. On the other hand, Asp331 is rather more conserved than Asp333 (Additional file 2: Figure S1). In any case, the resulting hydroxyl ion would nucleophilically attack the C1 carbon involved in the scissile glycosidic bond bound to the catalytic centre. Glu272 would act as proton donor to the glycosidic oxygen thereby promoting leaving group departure. With such enzymatic mechanisms involving proton transfers, enzymes often evolve micro-environments that shift pKa values for catalytic amino acids away from typical values. Computational prediction of pKa perturbation forms the basis of the annotation of likely catalytic residues by the THEMATICS method [60]. We submitted the BT2193 structure to the POOL server which integrates THEMATICS and cavity analyses [61]. The three putative catalytic residues - Asp333, Asp331 and Glu272 - are at positions 1, 3 and 5, respectively, in the ranked prediction list. In the THEMATICS results alone (authors, personal communication), discounting the cavity analysis, they form a cluster of pKa-perturbed residues, hence representing a predicted active site, although only perturbation of the pKa of Glu272 would be necessarily expected for the mechanism as outlined.

## Discussion

Various lines of evidence indicate the general involvement of the GxGYxYP family in carbohydrate metabolism, most compellingly the grouping of all four *Bacteroides thetaiotamicron* GxGYxYP-encoding genes into PULs. These loci are particularly powerful manifestations of the genome context approach to function prediction since proximity data are backed up by extensive transcriptomic profiling [10].

The first family structure reported here allows a more specific function prediction for the GxGYxYP family to be made, namely glycoside hydrolase activity. This derives first from a strong prediction of the location of a functional site, with both conservation and geometric analyses pointing to a cleft lying between an N-terminal domain of unusual, internally repeated structure and a C-terminal barrel fold. Within the cleft three conserved acidic residues, each predicted to have a perturbed pKa value as commonly seen for catalytic residues [60], lie in a conformation similar to that seen at the catalytic site of a cellulase of unrelated overall fold. Although seen in other GH families, the C-terminal 7-stranded barrel domain in the GxGYxYP family bears its catalytic site in a completely different position on the fold: indeed one of the predicted GxGYxYP catalytic residues lies on the preceding N-terminal domain.

Predicting a precise substrate will likely require further experimental data. BT2193, whose structure was determined, resides in a PUL of unknown carbohydrate specificity. Two other GxGYxYP proteins lie in a PUL responsive to α-mannans, used experimentally to determine loci involved in degradation of mannose-rich cores of host N-glycans. The PUL, extending from BT3853 to BT3862, contains two other GH enzymes, one (BT3958) from family GH92, which was shown to function as an α-1,3-specific mannosidase [62], the other (BT3862) from GH99, which displays endo-α-1,2-mannosidase activity, releasing 1,3-mannobiose from yeast mannan [63]. Together, these enzymes would mediate removal of the terminal decorations of fungal mannans. However, additional hydrolases are required to remove the α-1,2-mannosidic linkages at the base of the side chains, the phosphoryl groups, the hydrolysis of the α-1,6-linked mannose backbone and the β-1,4-mannosidic and N-acetyl-glucosidic linkages presented in the inner N-glycan core. Conceivably, one or other of these bonds represents a target for at least some GxGYxYP proteins, with the geometric similarity favouring the β-linkages targeted by the analogous GH9 cellulases discussed above. This hypothesis, as well as explaining the non-essentiality of GxGYxYP proteins in α-mannan responsive PULs (some would target fungal α-mannans and not host N-glycans), could also explain their presence in PULs not responsive to that carbohydrate: those loci might degrade N-glycans sharing the common core but decorated with other, non-mannose rich chains. Analysis of the GxGYxYP proteins in the mannan PULs, however, has so far failed to identify catalytic activity (HJG personal communication).

Interestingly, genes for mannosidases of GH92 and GH38 families lie near those encoding GxGYxYP proteins in the *Caldithrix abyssi* genome too. This bacterium of uncertain classification was isolated from a deep-sea hydrothermal chimney sample and its limited characterisation [64] and unpublished genome leave open the question as to the extent of its exploitation of environmental polysaccharides. Conceivably, it could associate with animal-derived mannose-containing glycoproteins either released on death or through interaction with living animals.

## Conclusion

We provide strong evidence from a wide variety of bioinformatics techniques that the GxGYxYP family, highly over-represented in gut bacteria, constitutes a new class of Glycoside Hydrolases. Further work will be required to determined substrate profiles for members and thereby to explain the strong association with the gut microbiome.

## Methods

### Crystallisation and structure refinement

Genomic DNA from *B. thetaiotaomicron* VPI-5482 (ATCC No. 29148D-5) was obtained from the American Type Culture Collection (ATCC). Protein production and crystallization of BT2193 gene product was carried out by standard JCSG protocols [65]. The crystal was obtained using the vapor diffusion method in a sitting drop format where sitting drops composed of 100 nl protein solution mixed with 100 nl crystallization solution were equilibrated against a 50 μl reservoir at 293 K. The crystallization reagent consisted of 20% polyethylene glycol 3350, 0.2 M ammonium chloride. Ethylene glycol was added to the crystal as a cryoprotectant to a final concentration of 10% (v/v). Data were collected at wavelengths corresponding to the inflection and high energy remote of a selenium MAD (multi-wavelength anomalous dispersion) experiment at 100 K using a MARCCD 325 detector (Rayonix) at Stanford Synchrotron Radiation Lightsource (SSRL) beamline 9_2. The initial structure trace was obtained using an automatic data processing and structure determination pipeline developed at JCSG [66]. Data processing were carried out using XDS [67] and the statistics are presented in Table S1. The structure was determined by the MAD method using programs SHELX [68] and autoSHARP [69], and refinement was carried out using REFMAC5 [70]. The structure was validated using the JCSG Quality Control server (http://smb.slac.stanford.edu/jcsg/QC). Atomic coordinates and experimental structure factors to 1.25 Å resolution (PDB code: 3sgg) have been deposited in the Protein Data Bank (PDB; http://www.rcsb.org, [71]).

### Sequence analysis

GxGYxYP family members were obtained by an iterative jackhmmer database search [72,73] with an inclusion threshold of e = 0.0001 in UniRef100, a non-redundant subset of the UniProt knowledgebase [74]. They were aligned with MAFFT [75] and the resulting alignment visualised and manipulated with Jalview [76]. Full-length sequences were subjected to bootstrapped phylogenetic analysis using MEGA 5 [13]. Briefly, the evolutionary history was inferred using the Neighbor-Joining method [77] with the bootstrap consensus tree inferred from 500 replicates [78]. The evolutionary distances were computed using the Poisson correction method [79] and are in the units of the number of amino acid substitutions per site with all ambiguous positions removed for each sequence pair. The final tree was displayed and coloured using Interactive Tree of Life (iTOL) [80]. Conservation from the same alignment was mapped onto the structure of BT2193 using the ConSurf server [43,44]. Domain architectures were obtained from Pfam [15]. Genomic context was studied using MicrobesOnline [14] and protein families identified using Pfam and InterPro [16].

### Structure-based function prediction

DALI [81] was used for structural similarity searches of the PDB. The crystal structure was submitted to ProFunc [41] for several structure-based analyses. Searches for 3D structural motifs representing catalytic or other binding sites were done using SPRITE [52] and PINTS [53]. The POOL server [61] was used to predict catalytic residues by integrating analyses of structure cavities and perturbed predicted pKa values [60]. Structures were visualised with PyMOL (http://www.pymol.org/), which was also used for structure figures.

## Competing interests

The authors declare that they have no competing interests.

## Authors’ contributions

QX refined the structure. QX and YC reported on the crystallography. RYE carried out the domain and genome context analyses. RYE and DJR did the phylogenetic analysis. DJR did the structure-based function prediction. AG analysed over-representation in MetaHIT. RYE, DJR, AG and HJG interpreted and synthesised the results. All authors wrote the paper and approved the final draft.

## Supplementary Material

Additional file 1: Table S1 Data collection and refinement statistics (PDB code 3sgg). Click here for file

Additional file 2: Figure S1 Sequence alignment of selected GxGYxYP family proteins. Identical residues are shown on a red background, conserved residues are shown in red in open boxes. The secondary structure is shown above the alignment. The alignment was displayed using ESPript [82]. Click here for file
