# Supplementary material for: Structure- and context-based analysis of the GxGYxYP family reveals a new putative class of Glycoside Hydrolase
Source: BMC Bioinformatics. 2014 Jun 17;15:196. doi: 10.1186/1471-2105-15-196 (PMC4071793; doi:10.1186/1471-2105-15-196)
Supplement: Additional file 1: Table S1 — Data collection and refinement statistics (PDB code 3sgg). [file 1471-2105-15-196-S1.docx]

**Supplementary Table 1. Data collection and refinement statistics (PDB code 3sgg)**

| **Data collection** |  | | |
| --- | --- | --- | --- |
| Beamline | SSRL 11-1 | | |
| Space group/Unit cell | C2, a=161.9, b=49.4, c=71.4Å, β=114.5° | | |
| Data | λ_1_ MADSe-infl | λ_2_ MADSe-remo | λ_3_ MADSe-peak |
| Wavelength (Å) | 0.97941 | 0.91837 | 0.97904 |
| Resolution range (Å) | 28.5-1.32 | 28.5-1.25 | 28.5-1.33 |
| No. of observations | 433797 | 510975 | 353826 |
| No. of unique reflections | 119604 | 140636 | 117106 |
| Completeness (%)^a^ | 99.2 (99.9) | 99.2 (99.8) | 99.4 (99.8) |
| Mean I/σ ^a^ | 15.8 (2.8) | 14.4 (2.4) | 15.1 (1.9) |
| R_merge_ on I^a^ (%) | 5.30 (45.80) | 5.60 (55.00) | 6.20 (59.90) |
| R_meas_ on I^a^ (%) | 6.30 (54.00) | 6.60 (64.90) | 7.50 (72.20) |
| R_pim_ on I^a^ (%) | 3.30 (28.20) | 3.40 (34.00) | 4.10 (39.90) |
| Highest resolution shell (Å) | 1.39-1.32 | 1.32-1.25 | 1.40-1.33 |
| **Model and refinement statistics** | | | |
| Data used in refinement | | λ_2_ MADSe |  |
| No. of reflections (total) | | 140635 |  |
| No. of reflections (test) | | 7057 |  |
| Cutoff criterion | | \|F\|>0 |  |
| R_cryst_ (%) | | 15.1 |  |
| R_free_ (%) | | 17.6 |  |
| **Stereochemical parameters** | | | |
| Restraints (RMSD observed) | | | |
| Bond lengths (Å) | | 0.014 |  |
| Bond angles (°) | | 1.466 |  |
| MolProbity all atom clash score | | 4.01 |  |
| Ramachandran plot (%)^b^ | | 98.0 (0) |  |
| Rotamer outlier (%) | | 0.2 |  |
| Average isotropic B-value (Å^2^)^c^ | | 11.4 (15.7) |  |
| ESU based on R_free_ (Å) | | 0.043 |  |
| No. protein residues / chains | | 512/1 |  |
| Non-protein entities | | 5 GOL, 550 H_2_O |  |

^a^Highest resolution shell in parentheses.

^b^Percentage of residues in favored regions of Ramachandran plot (No. outliers in parenthesis).

^c^This value represents the total B that includes TLS and residual B components (Wilson B-value in parathesis).

ESU = Estimated Standard Uncertainty in coordinates.

R_merge_=Σ_hkl_Σ_i_|I_i_(hkl)-<I(hkl)>|/Σ_hkl_Σ_i_I_i_(hkl), R_meas_(redundancy-independent R_merge_) =Σ_hkl_[N_hkl_/(N_hkl_-1)]^1/2^Σ_i_|I_i_(hkl)-<I(hkl)>|/Σ_hkl_Σ_i_I_i_(hkl), and R_pim_(precision-indicating R_merge_)=Σ_hkl_[1/(N_hkl_-1)]^1/2^Σ_i_|I_i_(hkl)-<I(hkl)>|/Σ_hkl_Σ_i_I_i_(hkl).

R_cryst_ =Σ| |F_obs_|-|F_calc_| |/Σ|F_obs_|, where F_calc_ and F_obs_ are the calculated and observed structure factor amplitudes, respectively.

R_free_ = as for R_cryst_, but for 5.0% of the total reflections chosen at random and omitted from refinement.
